# Light attention predicts protein location from the language of life

**DOI:** 10.1093/bioadv/vbab035

**Published:** 2021-11-19

**Authors:** Hannes Stärk, Christian Dallago, Michael Heinzinger, Burkhard Rost

**Affiliations:** 1 Department of Informatics, Bioinformatics & Computational Biology—i12, TUM (Technical University of Munich), 85748 Munich, Germany; 2 TUM Graduate School, Center of Doctoral Studies in Informatics and its Applications (CeDoSIA), 85748 Munich, Germany; 3 Institute for Advanced Study (TUM-IAS), 85748 Munich, Germany; 4 TUM School of Life Sciences Weihenstephan (WZW), Freising, Germany

## Abstract

**Summary:**

Although knowing where a protein functions in a cell is important to characterize biological processes, this information remains unavailable for most known proteins. Machine learning narrows the gap through predictions from expert-designed input features leveraging information from multiple sequence alignments (MSAs) that is resource expensive to generate. Here, we showcased using embeddings from protein language models for competitive localization prediction without MSAs. Our lightweight deep neural network architecture used a softmax weighted aggregation mechanism with linear complexity in sequence length referred to as light attention. The method significantly outperformed the state-of-the-art (SOTA) for 10 localization classes by about 8 percentage points (Q10). So far, this might be the highest improvement of *just embeddings* over MSAs. Our new test set highlighted the limits of standard static datasets: while inviting new models, they might not suffice to claim improvements over the SOTA.

**Availability and implementation:**

The novel models are available as a web-service at http://embed.protein.properties. Code needed to reproduce results is provided at https://github.com/HannesStark/protein-localization. Predictions for the human proteome are available at https://zenodo.org/record/5047020.

**Supplementary information:**

Supplementary data are available at *Bioinformatics Advances* online.

## 1 Introduction

### 1.1 Prediction bridges gap between proteins with and without location annotations

Proteins are the machinery of life involved in all essential biological processes (Supplementary Appendix: Biological Background). Knowing where in the cell a protein functions, *natively*, i.e. its *subcellular location* or *cellular compartment* (for brevity, abbreviated by *location*), is important to unravel biological function (Nair and Rost, 2005; Yu *et al.*, 2006). Experimental determination of protein function is complex, costly and selection biased (Ching *et al.*, 2018). In contrast, protein sequences continue to explode (The UniProt Consortium, 2021). This increases the sequence-annotation gap between proteins for which only the sequence is known and those with experimental function annotations. Computational methods have been bridging this gap (Rost *et al.*, 2003), e.g. by predicting protein location (Almagro Armenteros *et al.*, 2017; Goldberg *et al.*, 2012, 2014; Savojardo *et al.*, 2018). The standard tool in molecular biology, namely homology-based inference (‘HBI’), accurately transfers annotations from experimentally annotated to sequence-similar un-annotated proteins. However, HBI is either unavailable or unreliable for most proteins (Goldberg *et al.*, 2014; Mahlich *et al.*, 2018). Machine-learning methods perform less well (lower precision) but are available for all proteins (high recall). The best methods use evolutionary information as computed from families of related proteins identified in multiple sequence alignments (‘MSAs’) as input (Almagro Armenteros *et al.*, 2017; Goldberg *et al.*, 2012; Nair and Rost, 2005). Although the marriage of evolutionary information and machine learning has influenced computational biology for decades (Rost and Sander, 1993), due to database growth, MSAs have become costly.

### 1.2 Protein language models better represent sequences

Recently, protein sequence representations (embeddings) have been learned from databases (Steinegger and Söding, 2018; The UniProt Consortium, 2021) using language models (‘LMs’) (Alley *et al.*, 2019; Bepler and Berger, 2019; Elnaggar *et al.*, 2021; Heinzinger *et al.*, 2019; Rives *et al.*, 2021) initially used in natural language processing (‘NLP’) (Devlin *et al.*, 2019; Peters *et al.*, 2018; Raffel *et al.*, 2020). Models trained on protein embeddings via transfer learning tend to be outperformed by approaches using MSAs (Heinzinger *et al.*, 2019; Rao *et al.*, 2019). However, embedding-based solutions can outshine HBI (Littmann *et al.*, 2021) and advanced protein structure prediction methods (Bhattacharya *et al.*, 2020; Rao *et al.*, 2020; Weißenow *et al.*, 2021). Yet, for location prediction, embedding-based models (Elnaggar *et al.*, 2021; Heinzinger *et al.*, 2019; Littmann *et al.*, 2021) remained inferior to the state-of-the-art (‘SOTA’) using MSAs, such as DeepLoc (Almagro Armenteros *et al.*, 2017).

In this work, we leveraged protein embeddings to predict cellular location without MSAs. We proposed a deep neural network architecture using light attention (LA) inspired by previous attention mechanisms (Bahdanau *et al.*, 2015).

## 2 Related work

The best previous predictions of location prediction combined HBI, MSAs and machine learning, often building prior expert-knowledge into the models. For instance, *LocTree2* (Goldberg *et al.*, 2012) implemented profile-kernel [support-vector machines (‘SVMs’)] (Cortes and Vapnik, 1995), which identified *k*-mers conserved in evolution and put them into a hierarchy of models inspired by cellular sorting pathways. *BUSCA* (Savojardo *et al.*, 2018) combined three compartment-specific SVMs based on MSAs (Pierleoni *et al.*, 2006; Savojardo *et al.*, 2017). *DeepLoc* (Almagro Armenteros *et al.*, 2017) used convolutions followed by a bidirectional long short-term memory (‘LSTM’) module (Hochreiter and Schmidhuber, 1997) employing the Bahdanau-Attention (Bahdanau *et al.*, 2015). Using the BLOSUM62 substitution metric (Henikoff and Henikoff, 1992) for fast and MSAs for slower, refined predictions, DeepLoc rose to become the SOTA. Embedding-based methods (Heinzinger *et al.*, 2019) have not yet consistently outperformed this SOTA, although *ProtTrans* (Elnaggar *et al.*, 2021), based on very large datasets, came close.

## 3 Methods

### 3.1 Data

#### 3.1.1 Standard setDeepLoc

Following previous work (Elnaggar *et al.*, 2021; Heinzinger *et al.*, 2019), we began with a dataset introduced by *DeepLoc* (Almagro Armenteros *et al.*, 2017) for training (13 858 proteins) and testing (2768 proteins). All proteins have experimental evidence for 1 of 10 location classes (nucleus, cytoplasm, extracellular space, mitochondrion, cell membrane, endoplasmatic reticulum, plastid, Golgi apparatus, lysosome/vacuole and peroxisome). The 2768 proteins making up the test set (dubbed *setDeepLoc*), had been redundancy reduced to the training set (but not to themselves), and thus share ≤30*%* pairwise sequence identity (‘PIDE’) and *E*-values ≤10^– 6^ to any sequence in training. To avoid overestimations by tuning hyperparameters, we split the DeepLoc training set into: training-only (9503 proteins) and validation sets (1158 proteins; ≤30*%* PIDE; Supplementary Appendix: Datasets).

#### 3.1.2 Novel *setHARD*

To catch over-fitting on a static standard dataset, we created a new independent test set from *SwissProt* (The UniProt Consortium, 2021). Applying the same filters as *DeepLoc* (only eukaryotes; all proteins ≥40 residues; no fragments; only experimental annotations) gave 5947 proteins. Using *MMseqs2* (Steinegger and Söding, 2017), we removed all proteins from the new set with ≥20*%* PIDE to any protein in any other set. Next, we mapped location classes from DeepLoc to SwissProt, merged duplicates, and removed multi-localized proteins (protein X both in class Y and Z). Finally, we clustered at ≥20*%* PIDE leaving only one representative of each cluster in the new, more challenging test set (dubbed *setHARD*; 490 proteins; Supplementary Appendix: Datasets).

### 3.2 Models

#### 3.2.1 Input embeddings

As input to the ‘LA’ architectures, we extracted *frozen* embeddings from protein language models (pLMs), i.e. without fine-tuning for location prediction (details below). We compared embeddings from five main and a sixth additional pre-trained pLMs (Table 1): (i) ‘*SeqVec*’ (Heinzinger *et al.*, 2019) is a bidirectional LSTM based on ELMo (Peters *et al.*, 2018) that was trained on UniRef50 (Suzek *et al.*, 2015). (ii) ‘*ProtBert*’ (Elnaggar *et al.*, 2021) is an encoder-only model based on BERT (Devlin *et al.*, 2019) that was trained on BFD (Steinegger and Söding, 2018). (iii) ProtT5-XL-UniRef50 (Elnaggar *et al.*, 2021) (for simplicity: ‘*ProtT5*’) is an encoder-only model based on T5 (Raffel *et al.*, 2020) that was trained on BFD and fine-tuned on Uniref50. (iv) ‘*ESM-1b*’ (Rives *et al.*, 2021) is a transformer model that was trained on UniRef50. (v) ‘*UniRep*’ (Alley *et al.*, 2019) is a multiplicative LSTM (mLSTM)-based model trained on UniRef50. (vi) Bepler&Berger (dubbed ‘*BB*’) is a bidirectional LSTM by Bepler and Berger (2019), which fused modeling the protein language with learning information about protein structure into a single pLM. Due to different training objectives, this pLM was expected suboptimal for our task. As results confirmed this expectation, we confined these to Supplementary Appendix: Additional Results.

**Table 1.** ‘Implementation’ details for SeqVec (Heinzinger *et al.*, 2019), ProtBert (Elnaggar *et al.*, 2021), ProtT5 (Elnaggar *et al.*, 2021), ESM-1b (Rives *et al.*, 2021), UniRep (Alley *et al.*, 2019) and BB (Bepler and Berger, 2019)

	SeqVec	ProtBert	ProtT5	ESM-1b	UniRep	BB
Parameters	93M	420M	3B	650M	18.2M	90M*
Dataset	UniRef50	BFD	BFD	UniRef50	UniRef50	Pfam
Sequences	33M	2.1B	2.1B	27M	27M	21M
Embed time (s)	0.03	0.06	0.1	0.09	2.1	0.1
Attention heads	0	16	32	20	0	0
Bits per float	32	32	16	32	32	32
Size (GB)	0.35	1.6	3.6	7.3	0.06	0.12

*Notes*: Estimates marked by *; differences in the number of proteins (*Sequences*) for the same set (*Dataset*) originated from versioning. The embedding time (in seconds) was averaged over 10 000 proteins taken from the PDB (Berman *et al.*, 2000) using the embedding models taken from *bio-embeddings* (Dallago *et al.*, 2021).

Frozen embeddings were preferred over fine-tuned embeddings as the latter previously did not improve (Elnaggar *et al.*, 2021) and consumed more resources/energy. *ProtT5* was instantiated at half-precision (float16 weights instead of float32) to ensure the encoder could fit on consumer graphical processing units (GPUs) with limited vRAM. Due to model limitations, for ESM-1b, only proteins with fewer than 1024 residues were used for training and evaluation (Supplementary Appendix: Datasets).

Embeddings for each residue (NLP equivalent: word) in a protein sequence (NLP equivalent: document) were obtained using the bio-embeddings software (Dallago *et al.*, 2021). For *SeqVec*, the per-residue embeddings were generated by summing the representations of each layer. For all other models, the per-residue embeddings were extracted from the last hidden layer. Finally, the inputs obtained from the pLMs were of size *d*_in_ × *L*, where *L* is the length of the protein sequence, while *d*_in_ is the size of the embedding.

#### 3.2.2 Implementation details

The LA models were trained using filter size *s* = 9, *d*_out_ = 1024, the Adam (Kingma and Ba, 2015) optimizer (learning rate 5 × 10^– 5^) with a batch size of 150, and early stopping after no improvement in validation loss for 80 epochs. We selected the hyperparameters via random search (Supplementary Appendix: Hyperparameters). Models were trained either on an Nvidia Quadro RTX 8000 with 48 GB vRAM or an Nvidia GeForce GTX 1060 with 6 GB vRAM.

#### 3.2.3 LA architecture

**Fig. 1.**
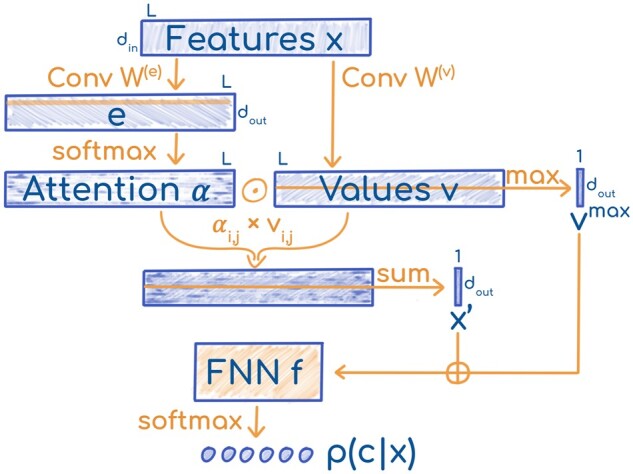
Sketch of LA. The LA architecture was parameterized by two weight matrices $W^{\left(e\right)},W^{\left(v\right)}\in R^{s\times d_{in}\times d_{\mathrm{out}}}$ and the weights of an FNN $f:R^{2d_{\mathrm{out}}}\mapsto R^{d_{\mathrm{class}}}$

The input to the ‘LA’ classifier (Fig. 1) was a protein embedding $x\in R^{d_{in}\times L}$ where *L* is the sequence length, while *d*_in_ is the size of the embedding (which depends on the model) (Table 1). The input was transformed by two separate 1D convolutions with filter sizes *s* and learned weights $W^{\left(e\right)},W^{\left(v\right)}\in R^{s\times d_{in}\times d_{\mathrm{out}}}$. The convolutions were applied over the length dimension to produce attention coefficients and values $e,v\in R^{d_{\mathrm{out}}\times L}$

(1) $$e_{i,j}=b_{i}+\sum_{k=1}^{d_{in}}\sum_{l=-\left\lfloor \frac{s}{2}\right\rfloor }^{\left\lceil \frac{s}{2}\right\rceil }W_{l,k,i}^{\left(e\right)}x_{k,j+l},$$

where $b\in R^{d_{\mathrm{out}}}$ is a learned bias. For j∉[0,L), the $x_{:,j}$ were zero vectors. To use the coefficients as attention distributions over all *j*, we *softmax-normalized them over the length dimension*, i.e. the attention weight $\alpha_{i,j}\in R$ for the *j*-th residue and the *i*-th feature dimension was calculated as:

(2) $$\alpha_{i,j}=\frac{\;\text{exp}(e_{i,j})}{\sum_{l=1}^{L}exp(e_{i,l})}.$$

Note that the weight distributions for each feature dimension *i* are independent, and they can generate different attention patterns. The attention distributions were used to compute weighted sums of the transformed residue embeddings $v_{i,j}$. Thus, we obtained a fixed-size representation $x^{\prime }\in R^{d_{\mathrm{out}}}$ for the whole protein, independent of its length.

(3) $$x_{i}^{\prime }=\sum_{j=1}^{L}\alpha_{i,j}v_{i,j},$$

(4) $$p(c|x)=\mathrm{softma}x_{c}(f(x^{\prime }⊕m)).$$

#### 3.2.4 Methods used for comparison

For comparison, we trained a two-layer feed-forward neural network (‘FNN’) proposed previously (Heinzinger *et al.*, 2019). Instead of per-residue embeddings in $R^{d_{in}\times L}$, the FNNs used sequence-embeddings in $R^{d_{in}}$, which derived from residue embeddings averaged over the length dimension (i.e. mean pooling). Furthermore, for these representations, we performed embeddings distance-based annotation transfer (dubbed ‘EAT’) (Littmann *et al.*, 2021). In this approach, proteins in *setDeepLoc* and *setHARD* were annotated by transferring the location from the nearest neighbor (L1 embedding distance) in the training set.

For ablations on the architecture, we tested LA without the softmax aggregation (‘LA w/o Softmax’) that previously produced $x^{\prime }$, by replacing it with averaging of the coefficients *e*. Then, with ‘LA w/o MaxPool’, we discarded the max-pooled values *v*^max^ as input to the FNN instead of concatenating them with $x^{\prime }$. With ‘Attention from *v*’, we computed the attention coefficients *e* via a convolution over the values *v* instead of over the inputs *x*. Additionally, we tested using a simple stack of convolutions (kernel-size 3, 9, and 15) followed by adaptive pooling to a length of 5 and an FNN instead of LA (‘Conv’ ± ‘AdaPool’). Similarly, ‘Query-Attention’ replaces the whole LA architecture with a transformer layer that used a single learned vector as query to summarize the whole sequence. As the last alternative operating on LM representations, we considered the ‘DeepLoc LSTM’ (Almagro Armenteros *et al.*, 2017) with *ProtT5* embeddings instead of MSAs (http://www.cbs.dtu.dk/services/DeepLoc).

To evaluate how traditional representations stack up against pLM embeddings, we evaluated MSAs [‘LA(MSA)’] and one-hot encodings of amino acids [‘LA(OneHot)’] as inputs to the LA model.

#### 3.2.5 Evaluation

Following previous work, we assessed performance through the mean 10-class accuracy (Q10), giving the percentage of correctly predicted proteins in 1 of 10 location classes. As additional measures tested [i.e. *F*1 score and Matthew correlation coefficient (MCC)] (Gorodkin, 2004) did not provide any novel insights, these were confined to the Supplementary Appendix: Additional Results. Error estimates were calculated over 10 random seeds on both test sets. For previous methods [DeepLoc and DeepLoc62 (Almagro Armenteros *et al.*, 2017), LocTree2 (Goldberg *et al.*, 2012), MultiLoc2 (Blum *et al.*, 2009), SherLoc2 (Briesemeister *et al.*, 2009), CELLO (Yu *et al.*, 2006), iLoc-Euk (Chou *et al.*, 2011), YLoc (Briesemeister *et al.*, 2010) and WoLF PSORT (Horton *et al.*, 2007)] published performance values were used (Almagro Armenteros *et al.*, 2017) for *setDeepLoc*. For *setHARD*, the webserver for DeepLoc (http://www.cbs.dtu.dk/services/DeepLoc) was used to generate predictions using either profile or BLOSUM inputs, whose results were later evaluated in Q10 and MCC. As a naive baseline, we implemented a method that predicted the same location class for all proteins, namely the one most often observed (in Results referred to as ‘Majority’). We provided code to reproduce all results (https://github.com/HannesStark/protein-localization).

## 4 Results

### 4.1 Embeddings outperformed MSAs

**Fig. 2.**
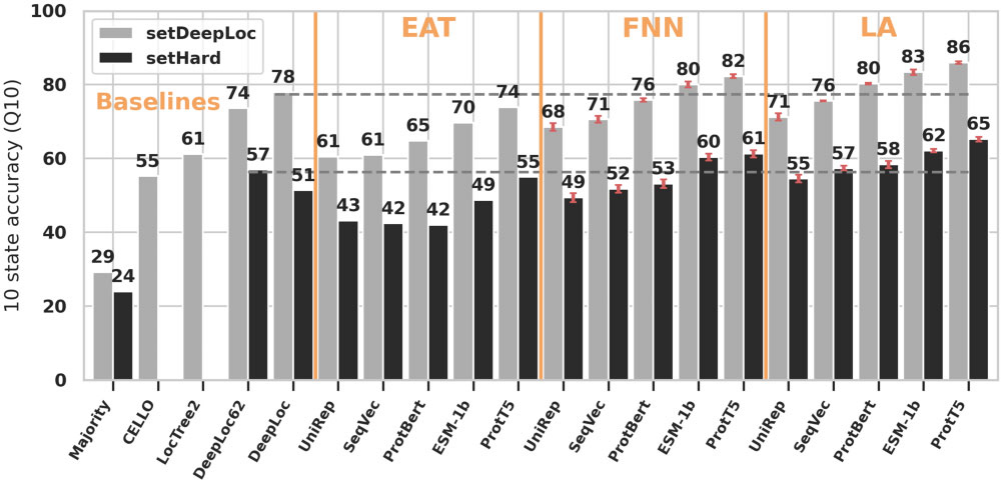
LA architectures performed best. ‘Performance’: bars give the 10-class accuracy (Q10) assessed on *setDeepLoc* (light-gray bars) and *setHARD* (dark-gray bars). ‘Methods’: *Majority*, CELLO*, LocTree2*, DeepLoc*, *DeepLoc62*; MSA-based methods marked by star. ‘EAT’ used the mean-pooled pLM embeddings to transfer annotation via distance, while ‘FNN(pLM)’ used the mean-pooled embeddings as input to a FNN. ‘LA(pLM)’ marked predictions using LA on top of the pLMs from: UniRep (Alley *et al.*, 2019), SeqVec (Heinzinger *et al.*, 2019), ProtBert (Elnaggar *et al.*, 2021), ESM-1b (Rives *et al.*, 2021) and ProtT5 (Elnaggar *et al.*, 2021). Horizontal gray dashed lines mark the previous SOTA (*DeepLoc* and *DeepLoc62)* on either set. Estimates for standard deviations are marked in red for the new methods. Overall, LA significantly outperformed the SOTA without using MSAs, and values differed substantially between the two datasets (light versus dark gray)

The simple embedding-based annotation transfer (EAT) already outperformed some advanced methods using MSAs (Fig. 2). The FNNs trained on *ProtT5* (Elnaggar *et al.*, 2021) and ESM-1b (Rives *et al.*, 2021) outperformed the SOTA *DeepLoc* (Almagro Armenteros *et al.*, 2017) (Fig. 2). Methods based on *ProtT5* embeddings consistently reached higher performance values than other embedding-based methods (*ProtT5 versus rest in Fig. 2). Results on Q10 were consistent with those obtained for MCC (Supplementary Appendix: Additional Results).

### 4.2 LA architecture best

The LA architecture introduced here consistently outperformed other embedding-based approaches for all pLMs tested (LA* versus EAT/FNN* in Fig. 2). Using *ProtBert* embeddings, LA outperformed the SOTA (Almagro Armenteros *et al.*, 2017) by 1 and 2 percentage points on *setHARD* and *setDeepLoc* [*LA(ProtBert)*Fig. 2]. For both test sets, LA improved the previous best on either set by around 8 percentage points with *ProtT5* embeddings.

### 4.3 Standard dataset over-estimated performance

The substantial drop in performance measures (by about 22 percentage points) between the standard *setDeepLoc* and the new challenging *setHARD* (Fig. 2: light-gray versus dark-gray, respectively) suggested substantial over-fitting. Mimicking the class distribution from *setDeepLoc* by sampling with replacement from *setHARD* led to higher values [Q10: *DeepLoc62* = 63%; *DeepLoc* = 54%; *LA(ProtBert)* = 62%; *LA(ProtT5)* = 69%)]. *DeepLoc* performed worse on *setHARD* with than without MSAs (only BLOSUM; Fig. 2: *DeepLoc* vs. *DeepLoc62*). Otherwise, the relative ranking and difference of models largely remained consistent between the two datasets *setDeepLoc* and *setHARD*.

### 4.4 Low performance for minority classes

**Fig. 3.**
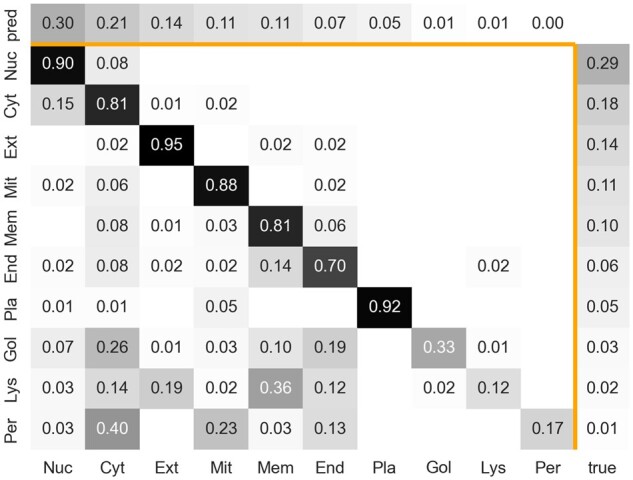
Mostly capturing majority classes. Confusion matrix of LA predictions on ProtT5 (Elnaggar *et al.*, 2021) embeddings for *setDeepLoc* (Almagro Armenteros *et al.*, 2017) (see Supplementary Appendix: Additional Results for setHARD). Darker color means higher fraction; the diagonal indicates accuracy for the given class; vertical axis: true class; horizontal axis: predicted class. Labels are sorted according to prevalence in ground truth with the most common class first (left or top). Labels: Nuc, **Nuc**leus; Cyt, **Cyt**oplasm; Ext, **Ext**racellular; Mit, **Mit**ochondrion; Mem, cell **Mem**brane; End, **End**oplasmatic Reticulum; Pla, **Pla**stid; Gol, **Gol**gi apparatus; Lys, **Lys**osome/vacuole; Per, **Per**oxisome; **pred**, distribution for predicted (proteins predicted in class X/total number of proteins); **true**, distribution for ground truth (proteins in class X/total number of proteins)

The confusion matrix of predictions for *setDeepLoc* using LA(*ProtT5*) highlighted how many proteins were incorrectly predicted to be in the second most prevalent class (*cytoplasm*), and that the confusion of the two most common classes mainly occurred between each other (Fig. 3: *nucleus* and *cytoplasm*). As for other methods, including the previous SOTA (Almagro Armenteros *et al.*, 2017), performance was particularly low for the most under-represented three classes (*Golgi apparatus*, *lysosome*/*vacuole* and *peroxisome*) that together accounted for 6% of the data. To attempt boosting performance for minority classes, we applied a *balanced loss*, assigning a higher weight to the contributions of under-represented classes. This approach did not raise accuracy for the minority classes but lowered the overall accuracy, thus it was discarded.

### 4.5 LA mechanism crucial

To probe the effectiveness of the LA aggregation mechanism on *ProtT5*, we considered several alternatives for compiling the attention (*LA w/o Softmax* & *LA w/o MaxPool* & *Attention from v* & *DeepLoc LSTM* & *Conv + AdaPool*), and used the LA mechanism with non-embedding input [*LA(OneHot)* & *LA(MSA)*]. Q10 dropped substantially without softmax- or max-aggregation. Furthermore, inputting *traditional* protein representations (*one-hot encoding*, i.e. representing the 20 amino acids by a 20-dimensional vector with 19 zeroes) or MSAs, the LA approach did not reach the heights of using pLM embeddings [Table 2: *LA(OneHot)* & *LA(MSA)*].

**Table 2.** Comparison of ‘LA(ProtT5)’ to different architectures and inputs

Method	setDeepLoc	setHARD
LA(ProtT5)	**86.0** ± 0.3	**65.2** ± 0.6
LA w/o Softmax	85.3 ± 0.3	64.7 ± 0.7
LA w/o Maxpool	84.7 ± 0.2	63.8 ± 0.7
Attention from *v*	85.4 ± 0.3	64.7 ± 0.9
DeepLoc LSTM	79.4 ± 0.9	59.3 ± 0.8
Conv + AdaPool	82.0 ± 0.9	60.7 ± 2.0
Query-Attention	75.3 ± 0.5	52.41 ± 0.4
LA(OneHot)	43.5 ± 1.5	32.5 ± 2.4
LA(MSA)	43.7 ± 1.3	33.3 ± 1.8

*Note*: Methods described in Section 3.2. Standard deviations are estimated from 10 runs with different weight initializations. The best performing method is highlighted in bold.

### 4.6 Model trainable on consumer hardware

Extracting ProtT5 pLM embeddings for all proteins used for evaluation took 21 min on a single Quadro RTX 8000 with 48 GB vRAM. Once those input vectors had been generated, the final LA architecture, consisting of 19 million parameters, could be trained on an Nvidia GeForce GTX 1060 with 6 GB vRAM in 18 h or on a Quadro RTX 8000 with 48 GB vRAM in 2.5 h.

## 5 Discussion

### 5.1 LA predicting location: beyond accuracy, four observations for machine learning in biology

The LA approach introduced here constituted possibly the largest margin to date of pLM embeddings improving over SOTA methods using MSAs. Although this improvement might become crucial to revive location prediction, ultimately this work might become even more important for other lessons learned:

- The LA solution improved substantially over all previous approaches to aggregate per-residue embeddings into per-protein embeddings for predictions. Many protein function tasks require per-protein representations, e.g. predictions of Gene Ontology, Enzyme Classifications, binary protein–protein interactions (to bind or not), cell-specific and pathway-specific expression levels. Indeed, LA might help in several of these tasks, too.
- Although static, standard datasets (here the *DeepLoc* data) jumpstart advances and help in comparisons, they may become a trap for over-estimates of performance through over-fitting. Indeed, the substantial difference in performance between *setDeepLoc* and *setHARD* highlighted this effect dramatically. Most importantly, our results underlined that claims of the type ‘*method NEW better than SOTA*’ should not necessarily constitute wedges for advancing progress. For instance, *NEW* on *setStandard* reaching P(NEW)¿P(SOTA) does not at all imply that *NEW* outperformed SOTA. Instead, it might point more to *NEW* over-fitting *setStandard*.
- The new data*setHARD* also pointed to problems with creating too well-curated datasets, such as *setDeepLoc*: one aim in selecting a *good* dataset is to use only the most reliable experimental results. However, those might be available for only some subset of proteins with particular features (e.g. short, well-folded). Experimental data are already extremely biased for the classes of location annotated (Marot-Lassauzaie *et al.*, 2021). Cleaning up might even increase this bias and thereby limit the validity of prediction methods optimized on those data. Clearly, existing location data differ substantially from entire proteomes (Marot-Lassauzaie *et al.*, 2021).
- *setHARD* also demonstrated that, unlike the protein structure prediction problem (Jumper *et al.*, 2021), the location prediction problem remains unsolved: while Q10 values close to 90% for *setDeepLoc* might have suggested levels close to—or even above—the experimental error, *setHARD* revealed values of Q10 below 70%. In fact, while most proteins apparently mostly locate in one compartment, for others the multiplicity of locations is a key to their role. This issue of *travelers vs. dwellers*, implies that Q10 cannot reach 100% as long as we count only one class as correctly predicted for each protein, and if we dropped this constraint, we would open another complication (Marot-Lassauzaie *et al.*, 2021). In short, the new dataset clearly generated more realistic performance estimates.

### 5.2 LA beats pooling

**Fig. 4.**
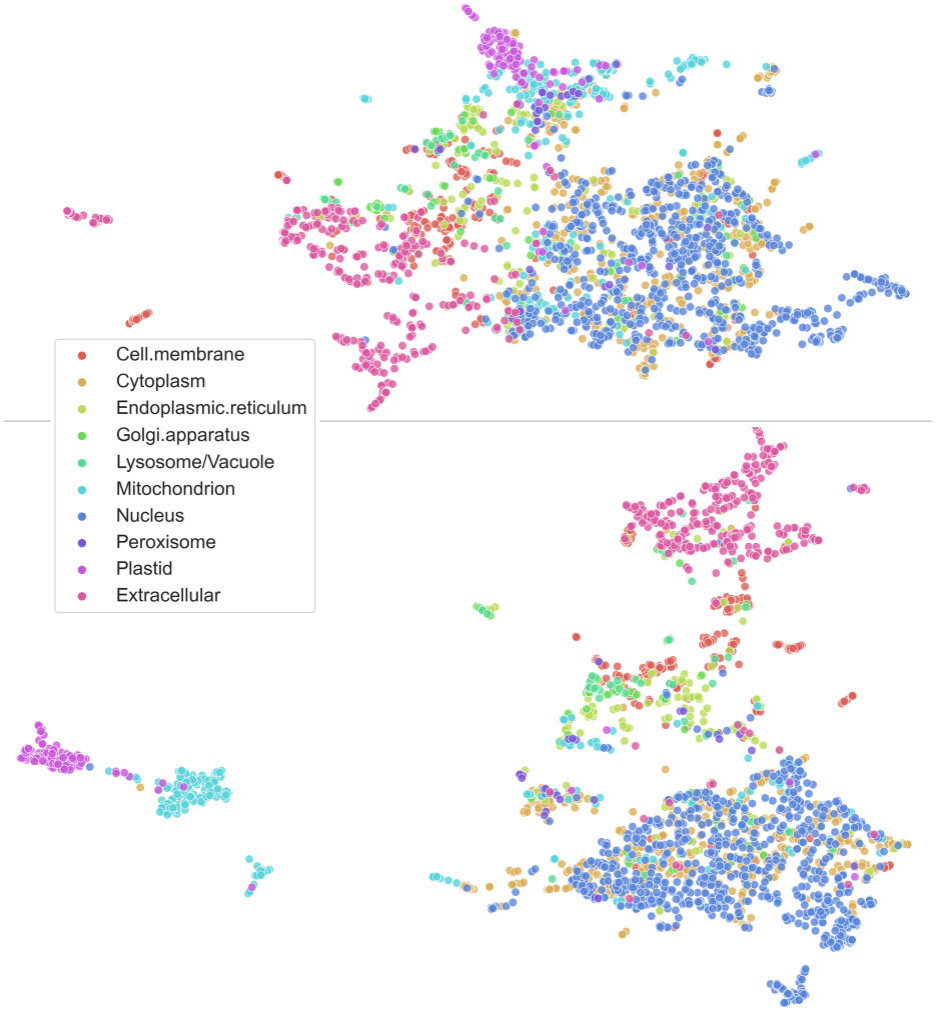
Qualitative analysis confirmed LA to be effective. UMAP (McInnes *et al.*, 2018) projections of per-protein embeddings colored according to subcellular location (*setDeepLoc*). Both plots were created with the same default values of the python *umap-learn* library. Top: ProtT5 embeddings (LA input; *x*) mean-pooled over protein length (as for FNN/EAT input). Bottom: ProtT5 embeddings (LA input; *x*) weighted according to the attention distribution produced by LA (this is not $x^{\prime }$ as we sum the input features *x* and not the values *v* after the convolution)

The central challenge for the improvement introduced here was to convert the per-residue embeddings (NLP equivalent: word embeddings) from pLMs [*BB* (Bepler and Berger, 2019), *UniRep* (Alley *et al.*, 2019), *SeqVec* (Heinzinger *et al.*, 2019), *ProtBert* (Elnaggar *et al.*, 2021), *ESM-1b* (Rives *et al.*, 2021) and *ProtT5* (Elnaggar *et al.*, 2021)] to meaningful per-protein embeddings (NLP equivalent: document). Qualitatively inspecting the influence of the LA mechanism through a UMAP comparison (Fig. 4) highlighted the basis for the success of the LA. The EAT surpassed some MSA-based methods without any optimization of the underlying pLMs (Fig. 2). In turn, inputting *frozen* pLM embeddings averaged over entire proteins into FNNs surpassed EAT and MSA-based methods (Fig. 2). The simple FNNs even improved over the SOTA, *DeepLoc*, for some pLMs (Fig. 2). However, LA consistently distilled more information from the embeddings. Most likely, the improvement can be attributed to LA coping better with the immense variation of protein length [varying from 30 to over 30 000 residues (The UniProt Consortium, 2021)] by learning attention distributions over the sequence positions. LA models appeared to have captured relevant long-range dependencies while retaining the ability to focus on specific sequence regions such as beginning and end, which play a particularly important role in determining protein location for some proteins (Almagro Armenteros *et al.*, 2017; Nair and Rost, 2005).

### 5.3 Embeddings outperformed MSA: first for function

Effectively, LA trained on pLM embeddings from *ProtT5* (Elnaggar *et al.*, 2021) was at the heart of the first method that clearly appeared to outperform the best existing method [*DeepLoc* (Almagro Armenteros *et al.*, 2017; Heinzinger *et al.*, 2019)] in a statistically significant manner on a new representative dataset not used for development (Fig. 2). To the best of our knowledge, it was also the first in outperforming the MSA-based SOTA in the prediction of subcellular location in particular, and of protein function in general. Although embeddings have been extracted from pLMs trained on large databases of un-annotated (unlabeled) protein sequences that evolved, the vast majority of data learned originated from much more generic constraints informative of protein structure and function. Clearly, pre-trained pLMs never had the opportunity to learn protein family constraints encoded in MSAs.

### 5.4 Better and faster than MSAs

When applying our solution to predict location for new proteins (or *at inference*), the embeddings needed as input for the LA models come with three advantages over the historically most informative MSAs that were essential for methods, such as *DeepLoc* (Almagro Armenteros *et al.*, 2017) to become top. Most importantly, embeddings can be obtained in far less time than is needed to generate MSAs and require fewer compute resources. Even the lightning-fast MMseqs2 (Steinegger and Söding, 2017), which is not the standard in bioinformatics (other methods 10–100× slower), in our experience, required about 0.3 s per protein to generate MSAs for a large set of 10 000 proteins. One of the slowest but most informative pLMs (*ProtT5*) is three times faster, while the third most informative (*ProtBert*) is five times faster (Table 1). Moreover, these MMseqs2 stats derive from runs on a machine with >300 GB of RAM and 2 ×40 cores/80threads CPUs, while generating pLM embeddings required only a moderate machine (8 cores, 16 GB RAM) equipped with a modern GPU with >7 GB of vRAM. Additionally, the creation of MSAs relied on tools, such as MMseqs2 that are sensitive to parameter changes, ultimately an extra complication for users. In contrast, generating embeddings required no parameter choice for users beyond the choice of the pLM (best here *ProtT5*). However, retrieving less specific evolutionary information [e.g. BLOSUM (Henikoff and Henikoff, 1992)] constituted a simple hash-table lookup. Computing such input could be instantaneous, beating even the fastest pLM *SeqVec*. Yet, these generic substitution matrices have rarely ever been competitive in predicting function (Bromberg *et al.*, 2008; Ng and Henikoff, 2003). One downside to use embeddings is the one-off expensive pLM pre-training (Elnaggar *et al.*, 2021; Heinzinger *et al.*, 2019). In fact, this investment pays off if and only if the resulting pLMs are not retrained. If they are used unchanged—as shown here—the advantage of embeddings over MSA is increasing with every single new prediction requested by users (over 3000/months just for *PredictProtein*) (Bernhofer *et al.*, 2021). In other words, every day, embeddings save more over MSAs.

### 5.5 Over-fitting through standard dataset?

For location prediction, the *DeepLoc* data (Almagro Armenteros *et al.*, 2017) has become a standard. Static standards facilitate method comparisons. To solidify performance estimates, we created a new test set (*setHARD*), which was redundancy reduced both with respect to itself and all proteins in the *DeepLoc* data (comprised of training plus testing data, the latter dubbed *setDeepLoc*). For *setHARD*, the 10-state accuracy (Q10) dropped, on average, 22 percentage points with respect to the static standard, *setDeepLoc* (Fig. 2). We argue that this large margin may be attributed to some combination of the following coupled effects.

- Previous methods may have been substantially overfitted to the static dataset, e.g. by misusing the test set to optimize hyperparameters. This could explain the increase in performance on *setHARD* when mimicking the class distributions in the training set and *setDeepLoc*.
- The static standard set allowed for some level of sequence-redundancy (information leakage) at various levels: certainly within the test set, which had not been redundancy reduced to itself (data not shown), maybe also between train and test set. Methods with many free parameters might more easily exploit such residual sequence similarity for prediction because proteins with similar sequences locate in similar compartments. In fact, this may explain the somewhat surprising observation that *DeepLoc* appeared to perform worse on *setHARD* using MSAs than the generic BLOSUM62 (Fig. 2: *DeepLoc62* vs. *DeepLoc*). Residual redundancy is much easier to capture by MSAs than by BLOSUM (Henikoff and Henikoff, 1992) (for computational biologists: the same way in which PSI-BLAST can outperform pairwise BLAST (Altschul *et al.*, 1997)].
- The confusion matrix (Fig. 3) demonstrated how classes with more experimental data tended to be predicted more accurately. As *setDeepLoc* and *setHARD* differed in their class composition, even without over-fitting and redundancy, prediction methods would perform differently on the two. In fact, this can be investigated by recomputing the performance on a similar class-distributed superset of *setHARD*, on which performance dropped only by 11, 24, 18 and 17 percentage points for *DeepLoc62*, *DeepLoc*, *LA(ProtBert)* and *LA(ProtT5)*, respectively.

Possibly, several effects contributed to the performance from standard to new dataset. Interestingly, different approaches behaved alike: both for alternative inputs from pLMs (*SeqVec*, *ProtBert* and *ProtT5*) and for alternative methods (EAT, FNN and LA), of which one (EAT) refrained from weight optimization.

### 5.6 What accuracy to expect for the next 10 location predictions?

If the top accuracy for one dataset was Q10 ∼60% and Q10 ∼80% for the other, what could users expect for their next 10 queries: either 6 correct or 8, or between 6 and 8? The answer depends on the query: if those proteins were sequence similar to proteins with known location (case: redundant): the answer would be eight. Conversely, for new proteins (without homologs of known location), 6 in 10 will be correctly predicted, on average. However, this assumes that the 10 sampled proteins follow somehow similar class distributions to what has been collected until today. In fact, if we applied *LA(ProtT5)* to a hypothetical new proteome similar to existing ones, we can expect the distribution of proteins in different location classes to be relatively similar (Marot-Lassauzaie *et al.*, 2021). Either way, this implies that for novel proteins, there seems to be significant room for pushing performance to further heights, possibly by combining *LA(ProtBert)*/*LA(ProtT5)* with MSAs.

## 6 Conclusion

We presented a LA mechanism in an architecture operating on embeddings from several pLMs (*BB*, *UniRep*, *SeqVec*, *ProtBert*, *ESM-1b* and *ProtT5*). LA efficiently aggregated information and coped with arbitrary sequence lengths, thereby mastering the enormous range of proteins spanning from 30 to 30 000residues. By implicitly assigning a different importance score for each sequence position (each residue), the method succeeded in predicting protein subcellular location much better than methods based on simple pooling. More importantly, for three pLMs, LA succeeded in outperforming the SOTA without using MSA-based inputs, i.e. the single most important input feature for previous methods. This constituted an important breakthrough: although many methods had come close to the SOTA using embeddings instead of MSAs (Elnaggar *et al.*, 2021), none had ever overtaken as the methods presented here. Our best method, *LA(ProtT5)*, was based on the largest pLM, namely on *ProtT5* (Fig. 2). Many methods were assessed on a standard dataset (Almagro Armenteros *et al.*, 2017)]. Using a new, more challenging dataset (*setHARD*), the performance of all methods appeared to drop by around 22 percentage points. While class distributions and dataset redundancy (or homology) may explain some of this drop, over-fitting might have contributed more. Overall, the drop underlined that many challenges remain to be addressed by future methods. For the time being, the best method *LA(ProtT5)* is freely available via a webserver (embed.protein.properties) and as part of a high-throughput pipeline (Dallago *et al.*, 2021). Predictions for the human proteome are available via Zenodo https://zenodo.org/record/5047020.

## Funding

This work was supported by the Deutsche Forschungsgemeinschaft (DFG)—project number RO1320/4-1, by the Bundesministerium für Bildung und Forschung (BMBF)—project number 031L0168; and by the BMBF through the program ‘Software Campus 2.0 (TU München)’—project number 01IS17049.

*Conflict of Interest*: none declared.

## Supplementary Material

vbab035_Supplementary_Data

## References

[vbab035-B1] Alley E.C. et al (2019) Unified rational protein engineering with sequence-based deep representation learning. Nat. Methods, 16, 1315–1322. 31636460 10.1038/s41592-019-0598-1 PMC7067682

[vbab035-B2] Almagro Armenteros J.J. et al (2017) DeepLoc: prediction of protein subcellular localization using deep learning. Bioinformatics, 33, 3387–3395. 29036616 10.1093/bioinformatics/btx431

[vbab035-B3] Altschul S.F. et al (1997) Gapped BLAST and PSI-BLAST: a new generation of protein database search programs. Nucleic Acids Res., 25, 3389–3402. 9254694 10.1093/nar/25.17.3389 PMC146917

[vbab035-B4] Bahdanau D. et al (2015) Neural machine translation by jointly learning to align and translate. In: Bengio Y., LeCun Y. (eds) *3rd International Conference on Learning Representations, ICLR 2015*. *San Diego, CA, USA, May 7-9, 2015, Conference Track Proceedings*. http://arxiv.org/abs/1409.0473.

[vbab035-B5] Bepler T. , Berger B. (2019) Learning protein sequence embeddings using information from structure. arXiv:*1902.08661 [cs.LG]*. http://arxiv.org/abs/1902.08661. PMC12947628 41768824

[vbab035-B6] Berman H.M. et al (2000) The protein data bank. Nucleic Acids Res., 28, 235–242. 10592235 10.1093/nar/28.1.235 PMC102472

[vbab035-B7] Bernhofer M. et al (2021) PredictProtein—predicting protein structure and function for 29 years. Nucleic Acids Res., 49, W535–W540. 33999203 10.1093/nar/gkab354 PMC8265159

[vbab035-B8] Bhattacharya N. et al (2020) Single layers of attention suffice to predict protein contacts. bioRxiv, 2020.12.21.423882. https://www.biorxiv.org/content/10.1101/2020.12.21.423882v2.

[vbab035-B9] Blum T. et al (2009) MultiLoc2: integrating phylogeny and Gene Ontology terms improves subcellular protein localization prediction. BMC Bioinformatics, 10, 274. 19723330 10.1186/1471-2105-10-274 PMC2745392

[vbab035-B10] Briesemeister S. et al (2009) SherLoc2: a high-accuracy hybrid method for predicting subcellular localization of proteins. J. Proteome Res., 8, 5363–5366. 19764776 10.1021/pr900665y

[vbab035-B11] Briesemeister S. et al (2010) YLoc—an interpretable web server for predicting subcellular localization. Nucleic Acids Res., 38, W497–W502. 20507917 10.1093/nar/gkq477 PMC2896088

[vbab035-B12] Bromberg Y. et al (2008) SNAP predicts effect of mutations on protein function. Bioinformatics, 24, 2397–2398. 18757876 10.1093/bioinformatics/btn435 PMC2562009

[vbab035-B13] Ching T. et al (2018) Opportunities and obstacles for deep learning in biology and medicine. J. R. Soc. Interface, 15, 20170387. 29618526 10.1098/rsif.2017.0387 PMC5938574

[vbab035-B14] Chou K.-C. et al (2011) iLoc-Euk: a multi-label classifier for predicting the subcellular localization of singleplex and multiplex eukaryotic proteins. PLoS One, 6, e18258. 21483473 10.1371/journal.pone.0018258 PMC3068162

[vbab035-B15] Cortes C. , Vapnik V. (1995) Support-vector networks. Mach. Learn., 20, 273–297.

[vbab035-B16] Dallago C. et al (2021) Learned embeddings from deep learning to visualize and predict protein sets. Curr. Protoc., 1, e113. 33961736 10.1002/cpz1.113

[vbab035-B17] Devlin J. et al (2019) BERT: pre-training of deep bidirectional transformers for language understanding. In: Burstein, J. *et al*. (eds) *Proceedings of the 2019 Conference of the North American Chapter of the Association for Computational Linguistics: Human Language Technologies, NAACL-HLT 2019*, *Minneapolis, MN, USA, June 2-7*, *2019*, *Volume 1 (Long and Short Papers)*. pp. 4171–4186. Association for Computational Linguistics. 10.18653/v1/n19-1423.

[vbab035-B18] Elnaggar A. et al (2021) ProtTrans: towards cracking the language of lifes code through self-supervised deep learning and high performance computing. IEEE Trans. Pattern Anal. Mach. Intell. doi: 10.1109/TPAMI.2021.3095381.

[vbab035-B19] Goldberg T. et al (2012) LocTree2 predicts localization for all domains of life. Bioinformatics, 28, i458–i465. 22962467 10.1093/bioinformatics/bts390 PMC3436817

[vbab035-B20] Goldberg T. et al (2014) LocTree3 prediction of localization. Nucleic Acids Res., 42, W350–W355. 24848019 10.1093/nar/gku396 PMC4086075

[vbab035-B21] Gorodkin J. (2004) Comparing two K-category assignments by a K-category correlation coefficient. Comput. Biol. Chem., 28, 367– 374. 15556477 10.1016/j.compbiolchem.2004.09.006

[vbab035-B22] Heinzinger M. et al (2019) Modeling aspects of the language of life through transfer-learning protein sequences. BMC Bioinformatics, 20, 723. 31847804 10.1186/s12859-019-3220-8 PMC6918593

[vbab035-B23] Henikoff S. , Henikoff J.G. (1992) Amino acid substitution matrices from protein blocks. Proc. Natl. Acad. Sci. USA, 89, 10915–10919. 1438297 10.1073/pnas.89.22.10915 PMC50453

[vbab035-B24] Hochreiter S. , Schmidhuber J. (1997) Long short-term memory. Neural Comput., 9, 1735–1780. 9377276 10.1162/neco.1997.9.8.1735

[vbab035-B25] Horton P. et al (2007) WoLF PSORT: protein localization predictor. Nucleic Acids Res., 35 (Suppl. 2), W585–W587. 17517783 10.1093/nar/gkm259 PMC1933216

[vbab035-B26] Jumper J. et al (2021) Highly accurate protein structure prediction with AlphaFold. Nature, 596, 583–589. 34265844 10.1038/s41586-021-03819-2 PMC8371605

[vbab035-B27] Kingma D.P. , Ba J. (2015) Adam: a method for stochastic optimization. In: Bengio Y. and LeCun Y. (eds) *3rd International Conference on Learning Representations, ICLR 2015, San Diego, CA, USA, May 7-9, 2015, Conference Track Proceedings*. http://arxiv.org/abs/1412.6980.

[vbab035-B28] Littmann M. et al (2021) Embeddings from deep learning transfer GO annotations beyond homology. Sci. Rep., 11, 1160. 33441905 10.1038/s41598-020-80786-0 PMC7806674

[vbab035-B29] Mahlich Y. et al (2018) HFSP: high speed homology-driven function annotation of proteins. Bioinformatics, 34, i304–i312. 29950013 10.1093/bioinformatics/bty262 PMC6022561

[vbab035-B30] Marot-Lassauzaie V. et al (2021) Spectrum of protein location in proteomes captures evolutionary relationship between species. J. Mol. Evol., 89, 544–553. 34328525 10.1007/s00239-021-10022-4 PMC8379119

[vbab035-B31] McInnes L. et al (2018) UMAP: uniform manifold approximation and projection. J. Open Source Softw., 3, 861.

[vbab035-B32] Nair R. , Rost B. (2005) Mimicking cellular sorting improves prediction of subcellular localization. J. Mol. Biol., 348, 85–100. 15808855 10.1016/j.jmb.2005.02.025

[vbab035-B33] Ng P.C. , Henikoff S. (2003) SIFT: predicting amino acid changes that affect protein function. Nucleic Acids Res., 31, 3812–3814. 12824425 10.1093/nar/gkg509 PMC168916

[vbab035-B34] Peters M. et al (2018) Deep contextualized word representations. In: *Proceedings of the 2018 Conference of the North American Chapter of the Association for Computational Linguistics: Human Language Technologies, Volume 1 (Long Papers)*. pp. 2227–2237. New Orleans, Louisiana, Association for Computational Linguistics. https://www.aclweb.org/anthology/N18-1202.

[vbab035-B35] Pierleoni A. et al (2006) BaCelLo: a balanced subcellular localization predictor. Bioinformatics, 22, e408–e416. 16873501 10.1093/bioinformatics/btl222

[vbab035-B36] Raffel C. et al (2020) Exploring the limits of transfer learning with a unified text-to-text transformer. J. Mach. Learn. Res., 21, 140:1–140:67.

[vbab035-B37] Rao R. et al (2019) Evaluating protein transfer learning with TAPE. Adv. Neural Inf. Process. Syst., 32, 9689–9701. 33390682 PMC7774645

[vbab035-B38] Rao R. et al (2020) Transformer protein language models are unsupervised structure learners. bioRxiv, 2020.12.15.422761. doi: 10.1101/2020. https://www.biorxiv.org/content/10.1101/2020.12.15.422761v1.

[vbab035-B39] Rives A. et al (2021) Biological structure and function emerge from scaling unsupervised learning to 250 million protein sequences. Proc. Natl. Acad. Sci. USA, 118, e2016239118. 33876751 10.1073/pnas.2016239118 PMC8053943

[vbab035-B40] Rost B. , Sander C. (1993) Prediction of protein secondary structure at better than 70% accuracy. J. Mol. Biol., 232, 584–599. 8345525 10.1006/jmbi.1993.1413

[vbab035-B41] Rost B. et al (2003) Automatic prediction of protein function. Cell. Mol. Life Sci., 60, 2637–2650. 14685688 10.1007/s00018-003-3114-8 PMC11138487

[vbab035-B42] Savojardo C. et al (2017) SChloro: directing Viridiplantae proteins to six chloroplastic sub-compartments. Bioinformatics, 33, 347–353. 28172591 10.1093/bioinformatics/btw656 PMC5408801

[vbab035-B43] Savojardo C. et al (2018) BUSCA: an integrative web server to predict subcellular localization of proteins. Nucleic Acids Res., 46, W459–W466. 29718411 10.1093/nar/gky320 PMC6031068

[vbab035-B44] Steinegger M. , Söding J. (2017) MMseqs2 enables sensitive protein sequence searching for the analysis of massive data sets. Nat. Biotechnol., 35, 1026–1028. 29035372 10.1038/nbt.3988

[vbab035-B45] Steinegger M. , Söding J. (2018) Clustering huge protein sequence sets in linear time. Nat. Commun., 9, 2542. 29959318 10.1038/s41467-018-04964-5 PMC6026198

[vbab035-B46] Suzek B.E. et al; the UniProt Consortium. (2015) UniRef clusters: a comprehensive and scalable alternative for improving sequence similarity searches. Bioinformatics, 31, 926–932. 25398609 10.1093/bioinformatics/btu739 PMC4375400

[vbab035-B47] The UniProt Consortium. (2021) UniProt: the universal protein knowledgebase in 2021. Nucleic Acids Res., 49, D480–D489. 33237286 10.1093/nar/gkaa1100 PMC7778908

[vbab035-B48] Weißenow K. et al (2021) Protein language model embeddings for fast, accurate, alignment-free protein structure prediction. bioRxiv: The Preprint Server for Biology. https://www.biorxiv.org/content/early/2021/08/02/2021.07.31.454572. 10.1016/j.str.2022.05.001 35609601

[vbab035-B49] Yu C.-S. et al (2006) Prediction of protein subcellular localization. Proteins, 64, 643–651. 16752418 10.1002/prot.21018

