# Supplementary material for: Light attention predicts protein location from the language of life
Source: Bioinform Adv. 2021 Nov 19;1(1):vbab035. doi: 10.1093/bioadv/vbab035 (PMC9710637; doi:10.1093/bioadv/vbab035)
Supplement: vbab035_Supplementary_Data [file vbab035_supplementary_data.pdf]

Appendix: Light Attention Predicts Protein Location from the Language of Life

Hannes Stärk [*1] Christian Dallago [*12] Michael Heinzinger [12] Burkhard Rost [134]

1. Protein Preliminaries

Protein Sequences. Proteins are built by chaining and arbitrary number of one of 20 amino acids in a particular order. When amino acids come together to form protein sequences, they are dubbed residues. During the assembly in the cell, constrained by physiochemical forces, the one-dimensional chains of residues fold into unique 3D shapes based solely on their sequence that largely determine protein function. The ideal machine learning model would predict a protein's 3D shape and thus function from just protein sequence (the ordered chain of residues).

Protein Subcellular Location. Eukaryotic cells contain different organelles/compartments. Each organelle serves a purpose, e.g., ribosomes chain together new proteins while mitochondria synthesize ATP. Proteins are the machinery used to perform these functions, including transport in and out and communication between different organelles and a cell's environment. For some compartments, e.g., the nucleus, special stretches of amino acids, e.g., nuclear localization signals (NLS), help identifying a protein's location via simple string matching. However, for many others, the localization signal is diluted within the whole sequence, requiring sequence-level predictions. Furthermore, some organelles (and the cell itself) feature membranes with different biochemical properties than the inside or outside, requiring protein gateways.

Homology-inference. Two highly similar protein sequences will most likely fold in similar 3D structures and more likely to perform similar functions. Homology based inference (Nair & Rost, 2002; Mahlich et al., 2018), which transfers annotations of experimentally validated proteins to query protein sequences, is based on this assumption (Sander & Schneider, 1991). Practically this means searching a database of annotated protein sequences for sequences that meet both an identity threshold and a length-of-match threshold to some query protein sequence. Sequence homology delivers good results, but its stringent requirements render it applicable to only a fraction of proteins (Rost, 1999).

Machine learning Function Prediction. When moving into territory where sequence similarity is less conserved for shorter stretches of matching sequences (Mahlich et al., 2018; Rost, 2002), one can try predicting function using evolutionary information and machine learning (Goldberg et al., 2012; Almagro Armenteros et al., 2017). Evolutionary information from protein profiles, encoding a protein's evolutionary path, is obtained by aligning sequences from a protein database to a query protein sequence and computing conservation metrics at the residue level. Using profiles leads to impressively more accurate predictions for sequences with no close homologs and has been the standard for most protein prediction tasks (Urban et al., 2020), including subcellular localization (Goldberg et al., 2012; Almagro Armenteros et al., 2017; Savojardo et al., 2018). While profiles provide a strong and useful inductive bias, their information content heavily depends on a balance of the number of similar proteins (depth), the overall length of the matches (sequence coverage), the diversity of the matches (column coverage), and their generation is parameter sensitive.

2. Hyperparameters

The following describes the search space used to find hyperparameters of our final LA and FNN models. We performed random search over these parameters. The evaluated learning rates were in the range of $[5 \times 10^{-6}$ - $5 \times 10^{-3}]$. For the light attention architecture, we tried filter sizes $[3, 5, 7, 9, 11, 13, 15, 21]$ and hidden sizes $d_{out} \in [32, 128, 256, 512, 1024, 1500, 2048]$, as well as concatenating outputs of convolutions with different filter sizes. For the FNN, we searched over the hidden layer sizes $[16, 32, 64, 512, 1024]$, where 32 was the optimium. We maximized batch size to fit a Quadro RTX 8000 with 48GB vRAM, resulting in the batch size of 150. Note that the memory requirement is dependent on the size of the longest sequence in a batch. In the DeepLoc dataset, the longest sequence had 13 100 residues.

3. Additional Results

We provide results for both *setDeepLoc* (Table 4) and *setHARD* (Table 3) in tabular form, including the Matthew's Correlation Coefficients (MCC) and class unweighted F1 score.

Furthermore, in Figure 1 we find that the UMAP projections of x' are more similar to those of the attention coefficients pooled along the length dimension and show clear clusters. Meanwhile, the projections of v^{max} in Figure 2 are less informative even though the ablations showed that v_max is important for the performance of our architecture.

Notable is that for both projections there are some clear outliers with the localization Plastid that are mapped far away from all other projections.

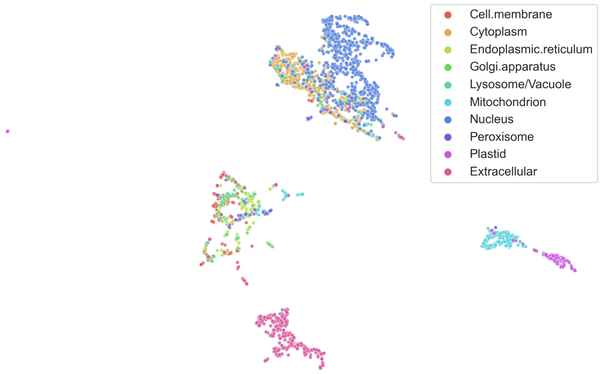

Figure 1. UMAP (McInnes et al., 2018) projections of x' embeddings colored according to subcellular location (*setDeepLoc*).

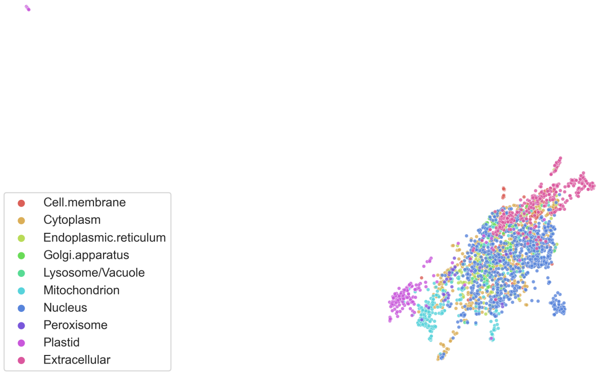

Figure 2. UMAP (McInnes et al., 2018) projections of v^{max} embeddings colored according to subcellular location (*setDeepLoc*).

Table 1. MCC of additional baselines and ablations compared to the LA architecture on *ProtT5* embeddings (above the line) of *setDeepLoc* and *setHARD* averaged over 10 seeds. The best method is **bold** and the second best is underlined.

METHOD	SETDEEPLOC	SETHARD
LA PROTT5	**.831**± .004	**.577**± .007
LA - SOFTMAX	.828± .004	.570± .008
LA - MAXPOOL	.816± .002	.559± .008
ATTENTION FROM V	.824± .003	.571± .012
DEEPLOC LSTM	.752± .010	.505± .009
CONV + ADAPOOL	.785± .010	.526± .022
MEANPOOL + FFN	.785± .006	.529± .010
LA ON ONEHOT	.326± .012	.216± .014
LA ON PROFILES	.302± .016	.195± .022

Table 2. Class unweighted F1 score of additional baselines and ablations compared to the LA architecture on *ProtT5* embeddings (above the line) of *setDeepLoc* and *setHARD* averaged over 10 seeds. The best method is **bold** and the second best is underlined.

METHOD	SETDEEPLOC	SETHARD
LA PROTT5	**.854**± .004	**.642**± .004
LA - SOFTMAX	.850± .004	.633± .008
LA - MAXPOOL	.842± .002	.632± .006
ATTENTION FROM V	.845± .004	.634± .011
DEEPLOC LSTM	.788± .009	.590± .007
CONV + ADAPOOL	.818± .010	.608± .020
MEANPOOL + FFN	.814± .005	.604± .008
LA ON ONEHOT	.367± .025	.262± .033
LA ON PROFILES	.384± .018	.279± .019

Table 3. Accuracy and Matthew's correlation coefficient (MCC) on *setHard*. Baseline= predict majority class; Evo= Previous methods based on evolutionary inputs; AT= assign class based on nearest neighbour in embedding space; FNN= predict using a Multi-Layer Perceptron on top of embeddings; LA= predict using LA on top of embeddings; Embedding inputs from: BB (Bepler & Berger, 2019), UniRep (Alley et al., 2019), SeqVec (Heinzinger et al., 2019), ProtBert (Elnaggar et al., 2021), ESM-1b (Rives et al., 2021), ProtT5 (Elnaggar et al., 2021).

	Method	Accuracy	MCC
	Baseline	24	0
Evo	DeepLoc62	<u>56.94</u>	<u>0.476</u>
	DeepLoc	51.36	0.410
AT	BB	25.98	0.133
	UniRep	43.15	0.329
	SeqVec	42.43	0.315
	ProtBert	42.04	0.306
	ESM-1b	48.72	0.386
	ProtT5	55.01	0.454
FNN	BB	35.60± 2.34	0.247± 0.025
	UniRep	49.41± 1.21	0.391± 0.013
	SeqVec	51.71± 1.04	0.398± 0.013
	ProtBert	53.16± 1.19	0.429± 0.014
	ESM-1b	60.40± 0.94	0.518± 0.010
	ProtT5	61.27± 0.97	0.529± 0.010
LA	BB	40.80± 2.44	0.293± 0.027
	UniRep	54.56± 1.07	0.451± 0.011
	SeqVec	57.37± 0.64	0.468± 0.013
	ProtBert	58.36± 1.02	0.490± 0.012
	ESM-1b	62.12± 0.5	0.537± 0.004
	ProtT5	**65.21**± 0.61	**0.577**± 0.007

Table 4. Accuracy and Matthew's correlation coefficient (MCC) on *setDeepLoc*. Baseline= predict majority class; Evo= Previous methods based on evolutionary inputs; AT= assign class based on nearest neighbour in embedding space; FNN= predict using a Multi-Layer Perceptron on top of embeddings; LA= predict using LA on top of embeddings; Embedding inputs from: BB (Bepler & Berger, 2019), UniRep (Alley et al., 2019), SeqVec (Heinzinger et al., 2019), ProtBert (Elnaggar et al., 2021), ESM-1b (Rives et al., 2021), ProtT5 (Elnaggar et al., 2021).

	Method	Accuracy	MCC
	Baseline	29	0
Evo	LocTree2	61.20	0.525
	MultiLoc2	55.92	0.487
	SherLoc2	58.15	0.511
	YLoc	61.22	0.533
	CELLO	55.21	0.454
	iLoc-Euk	68.20	0.641
	WoLF PSORT	56.71	0.479
	DeepLoc62	73.60	0.683
	DeepLoc	<u>77.97</u>	<u>0.735</u>
AT	BB	40.94	0.295
	UniRep	60.54	0.519
	SeqVec	60.97	0.508
	ProtBert	64.85	0.567
	ESM-1b	69.67	48.72
	ProtT5	73.92	0.687
FNN	BB	48.43± 0.99	0.367± 0.011
	UniRep	68.49± 1.02	0.622± 0.011
	SeqVec	70.57± 0.93	0.636± 0.011
	ProtBert	75.88± 0.45	0.702± 0.006
	ESM-1b	80.02± 0.84	0.760± 0.009
	ProtT5	82.28± 0.51	0.786± 0.006
LA	BB	55.75± 0.89	0.462± 0.010
	UniRep	71.24± 0.96	0.654± 0.011
	SeqVec	75.63± 0.11	0.705± 0.002
	ProtBert	80.29± 0.21	0.762± 0.002
	ESM-1b	83.39± 0.76	0.8013± 0.009
	ProtT5	**86.01**± 0.34	**0.832**± 0.004

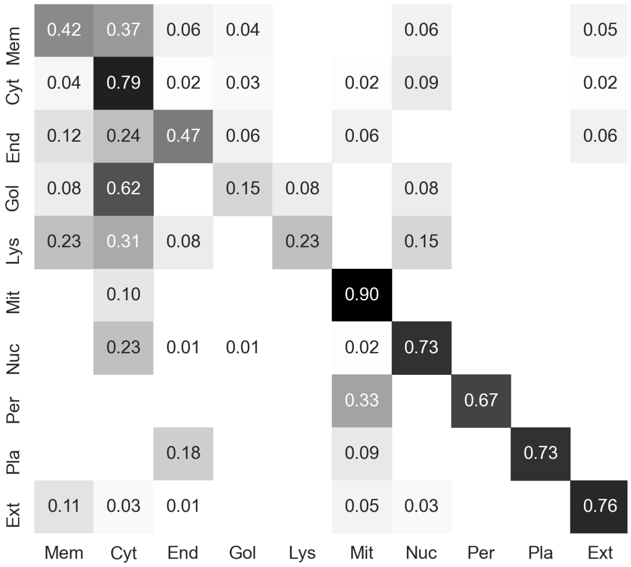

Figure 3. Confusion matrix of LA predictions on ProtT5 (Elnaggar et al., 2021) embeddings for *setHARD* annotated with the fraction of the true class. Vertical axis: true class, horizontal axis: predicted class. Labels: Mem=cell **Mem**brane; Cyt=**Cyt**oplasm; End=**End**oplasmatic Reticulum; Gol=**Gol**gi apparatus; Lys=**Lys**osome/vacuole; Mit=**Mit**ochondrion; Nuc=**Nuc**leus; Per=**Per**oxisome; Pla=**Pla**stid; Ext=**Ext**racellular

4. Datasets

Since ESM-1b can only process sequences shorter than 1024 residues, we removed the longer ones. This resulted in 8662 sequences for the training data, 2457 for *setDeepLoc*, and 431 for *setHard*. Table 5 shows the distribution of subcellular localization classes in the standard *setDeepLoc* and our new *setHARD* with all sequences included.

Table 5. Number of proteins and percentage of dataset for each class for the DeepLoc dataset and our *setHARD*. ER abbreviates Endoplasmatic Reticulum

LOCATION	DEEPLOC #	DEEPLOC %	SETHARD #	SETHARD %
NUCLEUS	4043	28.9	99	20.2
CYTOPLASM	2542	19.3	117	23.8
EXTRACELLULAR	1973	14.0	92	18.8
MITOCHONDRION	1510	11.8	10	2.0
CELL MEMBRANE	1340	9.5	98	20.0
ER	862	6.2	34	6.9
PLASTID	757	5.4	11	2.6
GOLGI APPARATUS	356	2.6	13	2.6
LYSOSOME/VACUOLE	321	2.3	13	2.2
PEROXISOME	154	1.1	3	0.6

4.1. New test set creation

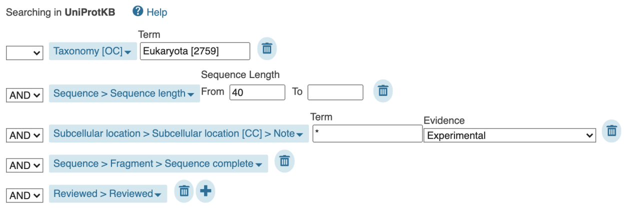

Figure 4. Screenshot of the filtering options applied to the advanced UniProt search (uniprot.org/uniprot).

In the following, we lay out the steps taken to produce the new test set (*setHARD*). The starting point is a filtered UniProt search with options as selected in Figure 4. Python code used is available at data.bioembeddings.com/public/data/new_test_set_procedure_code_data.zip.

- Download data as FASTA & XML:

```
wget "https://www.uniprot.org/
uniprot/?query=taxonomy:%
22Eukaryota%20[2759]%22%
20length:[40%20TO%20*]%
20locations:(note:*%20evidence:%
22Inferred%20from%20experiment%
20[ECO:0000269]%22)%20fragment:no%
20AND%20reviewed:yes&format=
xml&force=true&sort=score&compress=
yes"
```

```
wget "https://www.uniprot.org/
uniprot/?query=taxonomy:%
22Eukaryota%20[2759]%22%
20length:[40%20TO%20*]%
20locations:(note:*%20evidence:%
22Inferred%20from%20experiment%
20[ECO:000026%22)%20fragment:no%
20AND%20reviewed:yes&format=
fasta&force=true&sort=
score&compress=yes"
```

- Download deeploc data:

```
wget http://www.cbs.dtu.dk/services/
DeepLoc-1.0/deeploc_data.fasta
```

- Align sequences in swissprot to deeploc that have more than 20% PIDE:

```
mmseqs easy-search swissprot.fasta
deeploc_data.fasta -s 7.5
--min-seq-id 0.2 --format-output
query,target,fident,alnlen,mismatch,
gapopen,qstart,qend,tstart,tend,
evalue,bits,pident,nident,qlen,tlen,
qcov,tcov alignment.m8 tmp
```

- Extract localizations from SwissProt XML:

```
python extract_localizaiotns_from_
swissprot.py
```

- Map deeploc compartments on swissprot localizations & remove duplicates ([P123, Nucleus] appearing twice), remove multilocated ([P123, Nucelus] and [P123, Cytoplasm] –> remove P123) empty or not experimental annotations:

```
python map_and_filter_swissprot_
annotations.py
```

- Create FASTA like deeploc from sequences not in alignment:

```
python extract_unaligned_
sequences.py
```

- Redundancy reduce new set to 20%:

```
mmseqs easy-cluster --min-seq-id
0.2 new_test_set_not_redundancy_
reduced.fasta new_hard_test_set_
PIDE20.fasta tmp
```

References

Alley, E. C., Khimulya, G., Biswas, S., AlQuraishi, M., and Church, G. M. Unified rational protein

engineering with sequence-based deep representation learning. *Nature Methods*, 16(12):1315–1322, December 2019. ISSN 1548-7105. doi: 10.1038/s41592-019-0598-1. URL https://www.nature.com/articles/s41592-019-0598-1. Number: 12 Publisher: Nature Publishing Group.

Almagro Armenteros, J. J., Sønderby, C. K., Sønderby, S. K., Nielsen, H., and Winther, O. DeepLoc: prediction of protein subcellular localization using deep learning. *Bioinformatics*, 33(21):3387–3395, November 2017. ISSN 1367-4803. doi: 10.1093/bioinformatics/btx431. URL https://academic.oup.com/bioinformatics/article/33/21/3387/3931857.

Bepler, T. and Berger, B. Learning protein sequence embeddings using information from structure. *arXiv:1902.08661 [cs, q-bio, stat]*, October 2019. URL http://arxiv.org/abs/1902.08661. arXiv: 1902.08661.

Elnaggar, A., Heinzinger, M., Dallago, C., Rehawi, G., Yu, W., Jones, L., Gibbs, T., Feher, T., Angerer, C., Steinegger, M., Bhowmik, D., and Rost, B. ProtTrans: Towards Cracking the Language of Lifes Code Through Self-Supervised Deep Learning and High Performance Computing. *IEEE Transactions on Pattern Analysis and Machine Intelligence*, pp. 1–1, 2021. doi: 10.1109/TPAMI.2021.3095381.

Goldberg, T., Hamp, T., and Rost, B. LocTree2 predicts localization for all domains of life. *Bioinformatics*, 28 (18):i458–i465, September 2012.

Heinzinger, M., Elnaggar, A., Wang, Y., Dallago, C., Nechaev, D., Matthes, F., and Rost, B. Modeling aspects of the language of life through transfer-learning protein sequences. *BMC Bioinformatics*, 20(1):723, December 2019. ISSN 1471-2105. doi: 10.1186/s12859-019-3220-8. URL https://doi.org/10.1186/s12859-019-3220-8.

Mahlich, Y., Steinegger, M., Rost, B., and Bromberg, Y. HFSP: high speed homology-driven function annotation of proteins. *Bioinformatics*, 34(13):i304–i312, July 2018. ISSN 1367-4803. doi: 10.1093/bioinformatics/bty262. URL https://doi.org/10.1093/bioinformatics/bty262.

McInnes, L., Healy, J., Saul, N., and Großberger, L. UMAP: Uniform Manifold Approximation and Projection. *J. Open Source Softw.*, 3(29):861, 2018. doi: 10.21105/joss.00861. URL https://doi.org/10.21105/joss.00861.

Nair, R. and Rost, B. Sequence conserved for subcellular localization. *Protein Science*, 11(12):2836–2847, 2002. ISSN 1469-896X. doi: https://doi.org/10.1110/ps.0207402. URL https://onlinelibrary.wiley.com/doi/abs/10.1110/ps.0207402.

Rives, A., Meier, J., Sercu, T., Goyal, S., Lin, Z., Liu, J., Guo, D., Ott, M., Zitnick, C. L., Ma, J., and Fergus, R. Biological structure and function emerge from scaling unsupervised learning to 250 million protein sequences. *Proceedings of the National Academy of Sciences*, 118(15), 2021. ISSN 0027-8424. doi: 10.1073/pnas.2016239118. URL https://www.pnas.org/content/118/15/e2016239118.

Rost, B. Twilight zone of protein sequence alignments. *Protein Engineering, Design and Selection*, 12(2):85–94, February 1999. ISSN 1741-0126. doi: 10.1093/protein/12.2.85. URL https://doi.org/10.1093/protein/12.2.85.

Rost, B. Enzyme Function Less Conserved than Anticipated. *Journal of Molecular Biology*, 318 (2):595–608, April 2002. ISSN 0022-2836. doi: 10.1016/S0022-2836(02)00016-5. URL http://www.sciencedirect.com/science/article/pii/S0022283602000165.

Sander, C. and Schneider, R. Database of homology-derived protein structures and the structural meaning of sequence alignment. *Proteins: Structure, Function, and Bioinformatics*, 9(1):56–68, 1991. ISSN 1097-0134. doi: https://doi.org/10.1002/prot.340090107. URL https://onlinelibrary.wiley.com/doi/abs/10.1002/prot.340090107.

Savojardo, C., Martelli, P. L., Fariselli, P., Profiti, G., and Casadio, R. BUSCA: an integrative web server to predict subcellular localization of proteins. *Nucleic Acids Research*, 46(W1):W459–W466, 2018. ISSN 0305-1048. doi: 10.1093/nar/gky320. URL https://doi.org/10.1093/nar/gky320.

Urban, G., Torrisi, M., Magnan, C. N., Pollastri, G., and Baldi, P. Protein profiles: Biases and protocols. *Computational and Structural Biotechnology Journal*, 18:2281 – 2289, 2020. ISSN 2001-0370. doi: https://doi.org/10.1016/j.csbj.2020.08.015. URL http://www.sciencedirect.com/science/article/pii/S2001037020303688.
